# Synergistic Effects of Temozolomide and Doxorubicin in the Treatment of Glioblastoma Multiforme: Enhancing Efficacy through Combination Therapy

**DOI:** 10.3390/molecules29040840

**Published:** 2024-02-14

**Authors:** Laxmi Dhungel, Mandy E. Rowsey, Cayla Harris, Drazen Raucher

**Affiliations:** Department of Cell and Molecular Biology, University of Mississippi Medical Center, Jackson, MS 39216, USA; dhungel61@gmail.com (L.D.); mrowsey@umc.edu (M.E.R.); charris1@umc.edu (C.H.)

**Keywords:** glioblastoma, temozolomide, doxorubicin, drug synergy

## Abstract

Glioblastoma multiforme (GBM), a grade IV (WHO classification) malignant brain tumor, poses significant challenges in treatment. The current standard treatment involves surgical tumor removal followed by radiation and chemotherapeutic interventions. However, despite these efforts, the median survival for GBM patients remains low. Temozolomide, an alkylating agent capable of crossing the blood–brain barrier, is currently the primary drug for GBM treatment. Its efficacy, however, is limited, leading to the exploration of combination treatments. In this study, we have investigated the synergistic effects of combining temozolomide with doxorubicin, a chemotherapeutic agent widely used against various cancers. Our experiments, conducted on both temozolomide-sensitive (U87) and -resistant cells (GBM43 and GBM6), have demonstrated a synergistic inhibition of brain cancer cells with this combination treatment. Notably, the combination enhanced doxorubicin uptake and induced higher apoptosis in temozolomide-resistant GBM43 cells. The significance of our findings lies in the potential application of this combination treatment, even in cases of temozolomide resistance. Despite doxorubicin’s inability to cross the blood–brain barrier, our results open avenues for alternative delivery methods, such as conjugation with carriers like albumin or local administration at the surgical site through a hydrogel application system. Our study suggests that the synergistic interaction between temozolomide and doxorubicin holds promise for enhancing the efficacy of glioblastoma treatment. The positive outcomes observed in our experiments provide confidence in considering this strategy for the benefit of patients with glioblastoma.

## 1. Introduction

Glioblastoma multiforme (GBM) is a grade IV (WHO classification) malignant brain tumor that comprises 60% of adult brain tumors [1,2]. The disease can occur at any age; however, it is more prevalent in the older population (median age-64 years) and in men than in women (1.6:1) [1,3,4]. Except for exposure to ionizing radiation, there are no other known environmental risk factors for the disease; however, loose associations are seen with exposure to some chemicals such as vinyl chloride, pesticides, etc. [3]. There is also increased risk of disease development for patients with genetic diseases such as neurofibromatosis type 1, tuberous sclerosis, and Li-Fraumeni syndrome [1,3]. The clinical presentation of the disease includes increased intracranial pressure (headaches) and seizures; however, these symptoms appear late when the disease is already well established [1,3,5,6]. Glioblastoma can be categorized as primary or secondary GBM based on whether it emerged without a previous existing lesion (primary) or advanced from a low-grade astrocytoma (secondary) [2,3]. The current treatment strategy for glioblastoma is surgical removal of the tumor followed by radiation and chemotherapeutic treatment with temozolomide [3]. However, due to its invasive nature, the complete surgical removal of the tumor may not be achieved, hence the remaining tumor cells can grow, leading to tumor reoccurrence [3]. The median survival for glioblastoma patients is very low despite treatment (14–16 months) [1]. Hence, there is an urgent need to develop novel approaches such as effective drug delivery, discoveries of new drugs, or novel utilization of current in-use drugs that can reduce tumor burden and, thus, enhance patients’ survival and quality of life.

Temozolomide is the current standard chemotherapeutic agent for treating patients with glioblastoma [7]. Its antitumor activity was discovered in 1987 and was approved by the FDA for the treatment of glioblastoma in 2005 [8]. It has the ability to cross the blood–brain barrier and remains the first-line drug for the treatment of patients with glioblastoma [8,9]. Temozolomide is an alkylating agent that methylates DNA to cause DNA mismatch [7]. It arrests cells at the G2/M phase and induces apoptosis to kill the tumor cells [7,9]. However, the overall survival of patients despite this treatment is very low due to higher resistance development toward the drug [7]. There are several mechanisms that confer resistance to temozolomide, such as DNA repair via O6-methylguanine-DNA methyltransferase (MGMT), drug efflux, and survival autophagy [9]. Hence, there is a need to develop other alternative strategies (such as combination treatment with other drugs/immunotherapies or variation in temozolomide dose intensities) to achieve higher efficacy in tumor reduction and enhance the survival of patients [7].

Doxorubicin is a widely used chemotherapeutic agent for the treatment of cancers such as leukemia, brain cancer, breast cancer, stomach cancer, etc. [10,11]. The drug was discovered in the 1960s and was approved for cancer treatment by the Food and Drug Administration (FDA) in 1974 [11]. It can enter several organs such as the liver, heart, and kidney; however, it cannot pass the blood–brain barrier [11]. The antitumor activity of doxorubicin is mainly due to its inhibition of topoisomerase II, intercalation of DNA, and formation of free radicals [11,12]. Doxorubicin is administered in high doses during chemotherapeutic treatment due to its poor distribution ability, and it has been associated with causing toxicities to several organs such as the heart, liver, kidney, etc. [11,12]. Another concern related to the use of doxorubicin is the development of resistance toward the drug due to several mechanisms such as alterations in topoisomerase II enzymes, drug efflux, and mutations in P53 proteins [12]. Hence, to mitigate these challenges related to the use of doxorubicin, there is a need for alternative approaches that can enhance effective drug delivery (e.g., use of drug delivery systems such as nanoparticles) or increase its efficacy (e.g., modification of doxorubicin or combination treatment with other drugs) for the treatment of cancer [12].

The discovery of new anti-cancer drugs (discovery–clinical trials–approval) is time-consuming and expensive [13]. Further, resistance development in cancer cells to anti-cancer drugs has made cancer treatment challenging [14]. Thus, there is a need for alternative approaches, such as combination therapy, that can utilize the current in-use drugs to make treatment more effective. Combination treatments often utilize a combination of different anti-cancer drugs that can be used at lower concentrations compared to their single components and that can target different cancer pathways, hence conferring several advantages that include higher efficacy, reduced toxicity, and lower drug resistance [15]. The higher efficacy (at least additive effect) of a combination treatment compared to its single component was first demonstrated by Frei et al. (1965) for the treatment of children with acute lymphocytic leukemia [13,16]. Several clinical trial studies have been conducted, testing the efficacy of combination treatments of temozolomide with other agents such as irinotecan, bevacizumab, cilengitide, and lomustine for the treatment of glioblastoma [17,18,19,20]. The combination treatment with irinotecan exhibited higher toxicity, whereas combination treatment with bevacizumab showed higher adverse events [17,18]. The combination treatment with cilengitide did not show any benefit [19]. The combination treatment with lomustine showed an increase in median overall survival with moderate toxicities [20]. Apart from toxicities and efficacy related to the use of each drug, consideration should be made about the interaction of these drugs used for combination treatments. If the interaction of drugs used for the combination treatment is either synergistic or additive, then higher efficacy can be obtained, and any adverse effects can be avoided. Thus, testing the interaction of these drugs used for combination treatments in vitro can provide higher confidence in using them for clinical trials and for patient treatment. Hence, in this study, we investigated and demonstrated the synergistic interaction of two anti-cancer drugs: temozolomide and doxorubicin, and the underlying mechanisms for the treatment of glioblastoma. Both drugs target different cancer pathways and, thus, can address resistance issues related to individual drugs. Further, the use of these drugs in reduced concentrations can help to mitigate toxicity issues despite showing higher efficacy in the treatment. Doxorubicin has very high potency in killing cancer cells, and thus combination treatment of this drug with temozolomide, a drug currently used for the treatment of glioblastoma, can provide advantages that can enhance tumor reduction and patients’ survival [21].

## 2. Results

### 2.1. Effect of Combination of Temozolomide and Doxorubicin on Cell Viability

**Figure 1:**
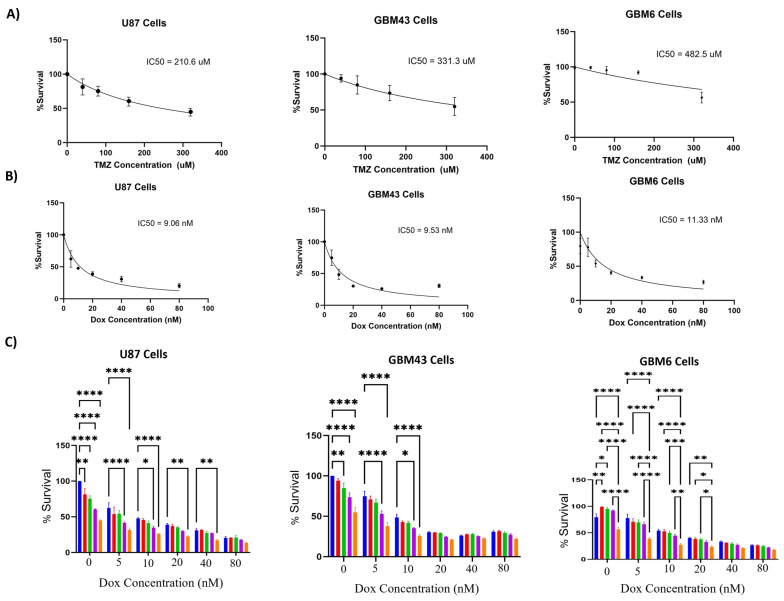
Effect of combination treatment with temozolomide and doxorubicin. (**A**) Temozolomide, (**B**) doxorubicin, and (**C**) combination treatment with temozolomide and doxorubicin in U87, GBM43, and GBM6 cells. The percentage survival for the combination treatment with temozolomide and doxorubicin was compared with the treatment with its single components. *p* values (* <0.05, ** <0.01, *** <0.001, **** <0.0001).

The percentage survival of cells treated with temozolomide or doxorubicin were determined for U87, GBM43, and GBM6 cell lines. The results showed that U87 cells were more sensitive to temozolomide compared to GBM43 and GBM6 cells (Figure 1). A significant decrease in percentage survival for U87 cells was observed for all the tested concentrations of temozolomide (40, 80, 160, 320 uM) compared to untreated cells, whereas a significant decrease in percentage survival was observed for GBM43 cells treated with temozolomide concentrations (80, 160, and 320 uM). Temozolomide (320 uM) caused more than 50% inhibition of U87 cells, whereas the same effect was not observed for GBM43 cells. For GBM6 cells, a significant decrease in percentage survival was observed for only temozolomide (320 uM). However, both (U87 and GBM43) cell lines were sensitive to doxorubicin (<50% survival for doxorubicin (10 nM)), whereas GBM6 had less than 50% survival with doxorubicin (20 nM). The percentage survival for all the cell lines was significantly lower (*p* < 0.05) when treated with the combination of doxorubicin (5, 10, 20, 40, and 80 nM) and temozolomide (40, 80, 160, 320 uM) compared to a single treatment with temozolomide at the same concentrations. Similarly, a significant decrease in the percentage survival of cell lines (U87 and GBM43) was observed for cells treated with a combination of doxorubicin (5 and 10 nM) and temozolomide (160 and 320 uM) compared to a single treatment with doxorubicin at the same concentrations. For GBM6 cells, a significant decrease in percentage survival was observed for cells treated with a combination of doxorubicin (5, 10 and 20 nM) and temozolomide (320 uM) compared to a single treatment with doxorubicin at the same concentrations.

### 2.2. Determination of Synergy between Temozolomide and Doxorubicin

**Figure 2:**
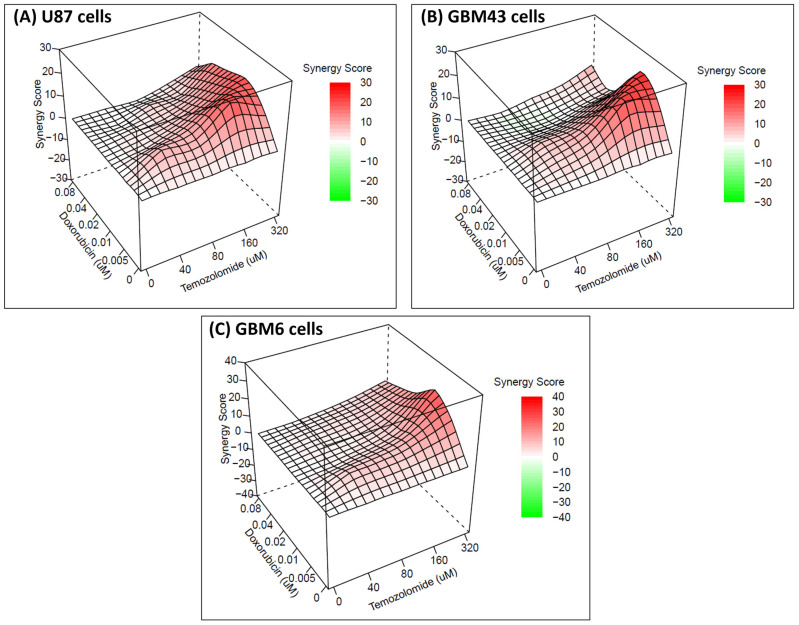
The 3D images showing synergy between the combination of drugs (temozolomide and doxorubicin) based on HSA score for different cell lines. (**A**) U87 cells, (**B**) GBM43 cells, and (**C**) GBM6 cells. The graph was generated using synergy finder, an online-based tool to determine synergy. Red color—synergistic, green color—antagonistic.

To test the synergy between the combination of drugs (temozolomide and doxorubicin), an online-based tool (synergy finder) was used (Figure 2). Synergy finder utilizes four different synergy reference models (HSA, Bliss, Loewe, and Zip) [22]. For U87 and GBM43 cells, neither synergy not antagonism was observed for the concentrations tested based on the Bliss, Loewe, and Zip models. However, based on the HSA model, synergy was observed for the combination of drugs at concentrations of temozolomide 160 uM–doxorubicin (5 and 10) nM and temozolomide 320 uM–doxorubicin (5, 10, 20, and 40 nM) for U87 cells. Similarly, the HSA model showed that the combination of drugs at concentrations of temozolomide 160 uM–doxorubicin (5 and 10) nM and temozolomide 320 uM–doxorubicin (5 and 10) nM is synergistic for GBM43 cells. For GBM6 cells, synergy was not observed for any concentrations tested based on the Bliss and Zip scores. Synergy was observed for the combination of drugs at temozolomide 320 uM–doxorubicin 80 nM based on the Loewe score. Based on the HSA score, synergy was observed for combination of drugs at concentrations of temozolomide 160 uM–doxorubicin 5 nM and temozolomide 320 uM–doxorubicin (5, 10, 20, and 40) nM.

### 2.3. Combination Treatment with Temozolomide Enhances Doxorubicin Uptake in GBM43 Cells

**Figure 3:**
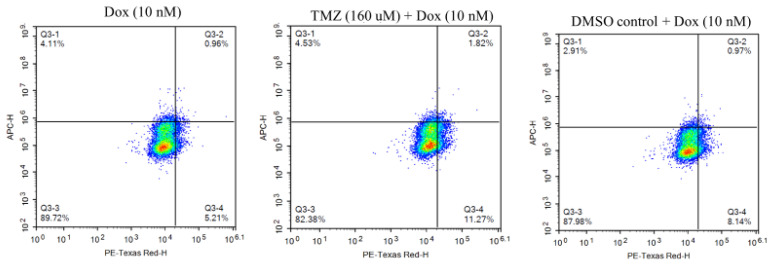
Representative image showing flow cytometry results for doxorubicin uptake. Cells that were detected on PE Texas red channel but not on APC channel (Q3-4) were considered positive for doxorubicin uptake.

**Figure 4:**
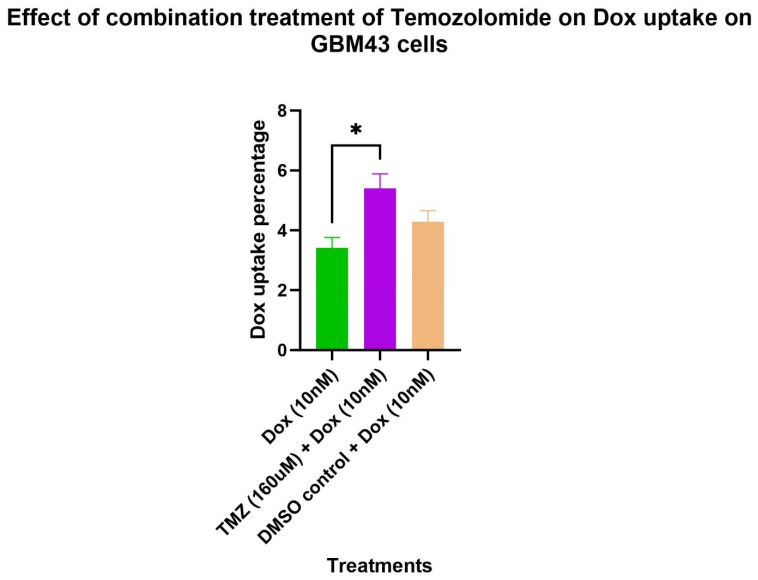
Effect of combination treatment of temozolomide and doxorubicin on doxorubicin uptake in GBM43 cells. The cells were treated with doxorubicin (10 nM) or combination treatment of temozolomide (160 uM) and doxorubicin (10 nM). Cells treated with DMSO and doxorubicin were used as solvent control for DMSO. The untreated cells were used for subtraction of autofluorescence from these treatment groups. The cells were stained with live/dead fixable Far Red Dead Cell Stain Kit to stain dead cells that could be detected on the APC channel. Doxorubicin has its own fluorescence and can be detected in the PE Texas Red channel. The percentage of cells detected on the PE Texas Red channel but not on the APC channel were considered to be positive for doxorubicin uptake. TMZ alone was not shown in the dox uptake experiment, as it does not generate any signal due to its non-fluorescent nature. The bar graph represents positive-cell percentages for doxorubicin uptake for each treatment group after subtraction of autofluorescence from untreated cells. *p* value (* <0.05).

To determine the effect of the combination treatment on doxorubicin uptake percentage, the cells were treated with either doxorubicin (10 nM), temozolomide (160 uM) + doxorubicin (10 nM), or DMSO control (equivalent volume used for temozolomide (160 uM) + doxorubicin (10 nM) (Figure 3). The untreated cells were used to subtract the autofluorescence. The cells were stained with a live/dead stain kit that stains dead cells and is detected on an APC channel. Doxorubicin fluorescence can be detected in the PE Texas Red channel. Hence, the percentage of cells detected on the PE Texas Red channel but not on the APC channel were measured to determine the doxorubicin uptake percentage. The result showed a significantly higher uptake percentage (1.6 times) in cells treated with temozolomide (160 uM) + doxorubicin (10 nM) compared to cells treated with doxorubicin (10 nM) alone (Figure 4). Similarly, there was a higher doxorubicin uptake percentage (1.26 times) in cells treated with temozolomide (160 uM) + doxorubicin (10 nM) compared to cells treated with DMSO (equivalent volume of temozolomide (160 uM)) + doxorubicin 10 nM; however, the doxorubicin uptake percentage was not statistically significant.

### 2.4. Effect of Combination Treatment of Temozolomide and Doxorubicin on Apoptosis

**Figure 5:**
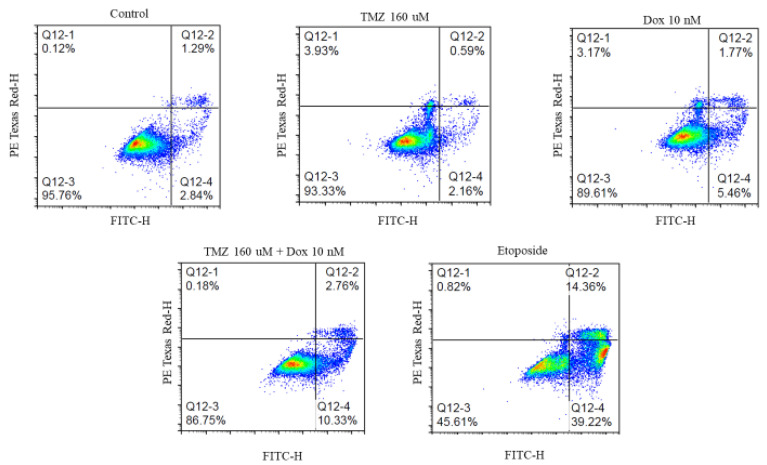
Effect of different treatments on apoptosis of GBM43 cells. The cells were stained with propidium iodide (PI) (detected on PE Texas Red channel) and annexin V (detected on FITC channel). The cells negative for both stains represent healthy cells (Q12-3), cells positive for annexin V but not PI represent early apoptosis (Q12-4), cells positive for both annexin V and PI represent late apoptosis (Q12-2), and the cells negative for annexin V but positive for PI represent necrosis (Q12-1).

**Figure 6:**
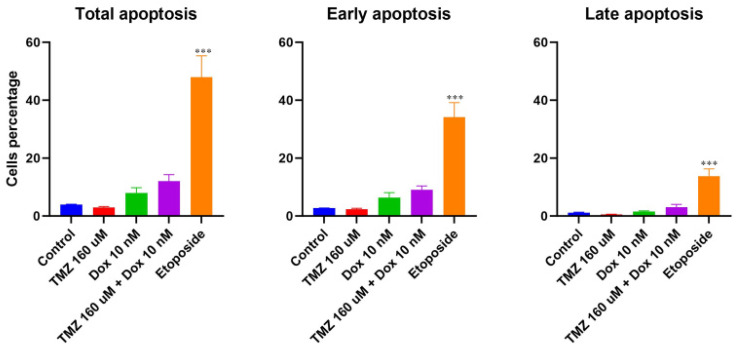
Line graphs showing total, early, and late apoptosis of GBM43 cells for different treatments. *p* value (*** <0.001).

The total, early, and late apoptosis of cells treated with a single treatment of temozolomide or doxorubicin were compared to apoptosis for cells treated with a combination treatment with temozolomide and doxorubicin (Figure 5). The cells treated with 0.34% DMSO were the negative control and cells treated with etoposide (500 uM) were the positive control. The results showed a significantly higher total, early, and late apoptosis in cells treated with etoposide compared to all the other treatments. The cells treated with the combination treatment of temozolomide and doxorubicin showed higher apoptosis compared to cells treated with a single treatment of either temozolomide or doxorubicin (4 times and 1.5 times, respectively). Although the apoptosis for the combination treatment was not significantly different based on one-way ANOVA analysis, the unpaired t-test comparing the combination treatment of temozolomide and doxorubicin with the treatment of temozolomide only showed statistically significant differences in total and early apoptosis (Figure 6). Similar statistically significant differences were not observed when comparing cells treated with combination treatment with cells treated with doxorubicin only.

### 2.5. Effect of Combination Treatment of Temozolomide and Doxorubicin on Cell Cycle

**Figure 7:**
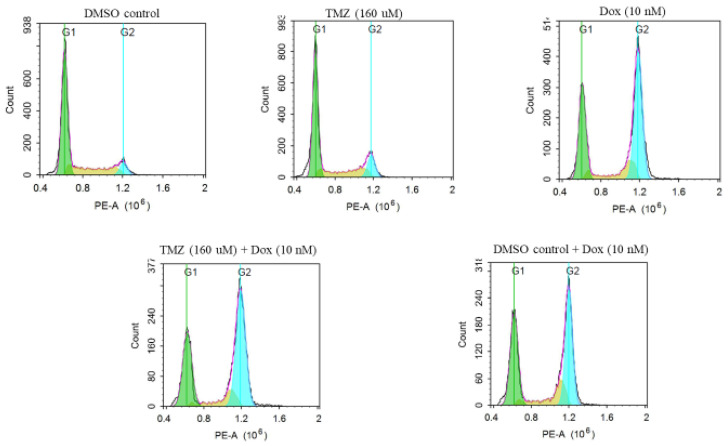
Representative image showing cell cycle arrest for different treatment groups. The cells were stained with PI and detected on PE channel on flowcytometry.

**Figure 8:**
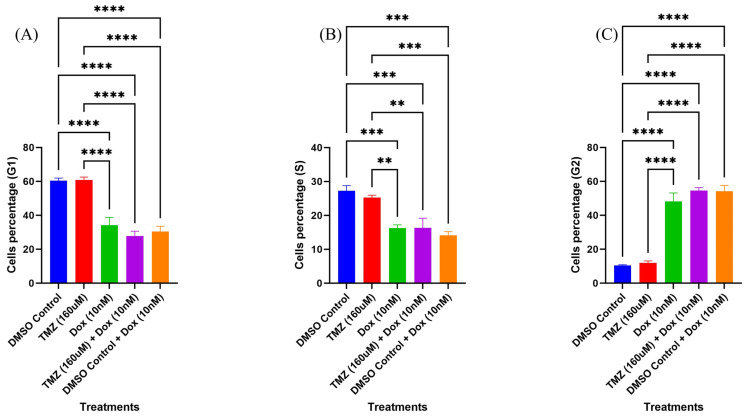
Effect of combination treatment of temozolomide and doxorubicin on cell cycle. (**A**) Cell cycle arrest for different treatment groups at the G1 phase, (**B**) cell cycle arrest for different treatment groups at the S phase, (**C**) cell cycle arrest for different treatment groups at the G2 phase. **** (*p* value < 0.0001), *** (*p* value < 0.001), ** (*p* value < 0.01).

The cell cycle analysis showed higher cell cycle arrest at the G1 phase for control (DMSO-treated) groups and cells treated with temozolomide only (Figure 7). However, the cells treated with doxorubicin, the combination treatment of temozolomide and doxorubicin, and the combination treatment of DMSO and doxorubicin showed higher cell cycle arrests at the G2 phase. The cell cycle arrests (indicated by percentage of cells at the G1, S, and G2 phases) were significantly different in cells treated with doxorubicin, the combination treatment of temozolomide and doxorubicin, and combination treatment of DMSO and doxorubicin compared to the control and the cells treated with temozolomide only (Figure 8). However, there was no significant difference in cell cycle arrest in any of the cells treated with doxorubicin (doxorubicin, combination treatment of temozolomide and doxorubicin, and combination treatment of DMSO and doxorubicin), suggesting the role of doxorubicin for arrest at the G2 phase. Similarly, there was no significant difference in cell cycle arrest for the control groups and cells treated with temozolomide only.

## 3. Discussion

In this study, we investigated the inhibitory effects of temozolomide, doxorubicin, and their combination treatment on three different cell lines (U87-temozolomide-sensitive, GBM6, and 43-temozolomide-resistant). Doxorubicin had a higher inhibitory effect against all these cell lines compared to temozolomide. There was almost 70% inhibition of GBM43 and U87 cells by doxorubicin at the 20 nM and 40 nM concentrations, respectively; however, a similar killing effect was not observed for any of the tested concentrations of temozolomide. For concentrations of doxorubicin below these inhibitory effect concentrations (almost 70% inhibition), a higher killing effect of the combination treatment was observed compared to treatment with doxorubicin at the same concentrations. Similarly, the combination treatment had a higher inhibitory effect on all of the cell lines compared to the treatment with temozolomide at the same concentrations. The combination treatment of temozolomide and doxorubicin was not antagonistic (synergy score < −10) for any of the concentrations tested, indicating that the combination of these drugs is safe to use for the treatment of glioblastoma. More specifically, combination treatment with higher concentrations of temozolomide (160 and 320 uM) and lower concentrations of doxorubicin (5 and 10 nM) can provide better treatment as synergy (synergy score > 10) was observed for these combinations for both U87 and GBM43 cells based on the HSA model. Previous studies by Baloi et al. (2022) and Ros et al. (2018) have also investigated combination treatment with temozolomide and doxorubicin on glioblastoma [23,24]. Baloi et al. (2022) have investigated the effects of combination therapy (temozolomide–doxorubicin) on GB2B cell lines and found no synergistic interaction between these drugs [23]. Similarly, Ros et al. (2018) have investigated the effect of combination treatment with doxorubicin-derivative (aldoxorubicin) and temozolomide on three different cells lines (U87MG, A172, and T98G) and demonstrated higher killing with the combination treatment of aldoxorubicin and temozolomide for temozolomide-resistant (T98G) cells, but a similar higher killing effect was not seen for other two cell lines [24]. However, in both of these studies the combination treatment included only one concentration for doxorubicin/aldoxorubicin and one or two concentrations for temozolomide [23,24]. Further, Baloi et al. (2022) compared predicted and observed survivals for the combination treatment, and Ros et al. (2018) compared significant, higher killing for the combination treatment compared to killing by its single component to determine the interaction between these drugs [23,24]. However, in our current study, we used a wider range of concentrations of both temozolomide and doxorubicin and used an online-based tool (Synergyfinder) that uses different models (HSA, Bliss, Loewe, and ZIP) to determine the synergy for these drugs. The results from our synergy study indicate that the combination treatment of temozolomide and doxorubicin can be safer (with no antagonistic effects) and have higher efficacy in inhibiting brain cancer cells.

Further, we conducted experiments on doxorubicin uptake, cell cycle, and apoptosis to elucidate the underlying mechanisms of the higher efficacy of the combination treatment of temozolomide and doxorubicin in GBM43 cells. The fluorescence ability of doxorubicin can be utilized to determine doxorubicin uptake by GBM43 cells. In our study, we found significantly higher doxorubicin uptake in cells treated with the combination of temozolomide and doxorubicin compared to cells treated with doxorubicin only. The solvent used for temozolomide had DMSO, which may facilitate the penetration of compounds into cells; however, the doxorubicin uptake on the cells treated with DMSO (equivalent volume used for temozolomide) did not show a similar effect, indicating that the higher doxorubicin uptake observed in the cells was mainly due to temozolomide treatment [25]. Treatment with temozolomide induces stress that has shown to induce genotypic, phenotypic, and metabolic changes in GBM cells [26,27]. Caragher et al. (2020) have observed a metabolic shift from glucose to lipid metabolism and higher fatty acid uptake in GBM cells treated with temozolomide [26]. Further, doxorubicin uptake is impacted by several factors such as pH (extracellular and intracellular) [28]. Although a conclusion on an exact mechanism for the higher doxorubicin uptake in cells treated with temozolomide cannot be drawn without further experiments, we can explain some of the possible mechanisms that could play role in the higher doxorubicin uptake in cells treated with temozolomide. Temozolomide treatment can induce certain physiologic changes in cells (such as pH level) that can enhance higher doxorubicin uptake. Further, temozolomide may increase membrane permeability, facilitating a higher penetration of doxorubicin. Despite the exact mechanism being unknown, the synergistic interaction of temozolomide–doxorubicin and higher doxorubicin uptake in GBM43 cells indicates that this approach of combination treatment can be applied to even temozolomide-resistant cells to obtain higher efficacy in the treatment of glioblastoma.

In the cell cycle experiment, we observed reduced cell cycle arrest at the G2 phase for cells treated with temozolomide only. Temozolomide causes cell cycle arrest at the G2/M phase; however, temozolomide-resistant cells can bypass this arrest [9,29]. Zhu et al. (2022) have observed a similar decrease in arrest at the G2/M phase for temozolomide-resistant cells [29]. There was higher cell cycle arrest at the G2 phase for cells treated with doxorubicin only. This result was as expected, as doxorubicin causes cell cycle arrest at the G2/M phase [30]. The cells treated with the combination treatment of temozolomide and doxorubicin also had higher cell cycle arrest at the G2 phase and had cell cycle arrest similar to the cells treated with doxorubicin only. Both cells treated with doxorubicin and cells treated with the combination treatment with temozolomide and doxorubicin had cell cycle arrests significantly that were different than the cells treated with temozolomide only. The difference in this cell cycle arrest could be mainly driven by doxorubicin, as single treatment with doxorubicin and the combination treatment with temozolomide and doxorubicin had similar cell cycle arrests at the G2 phase. Hence, the combination treatment with temozolomide does not contribute to enhancing cell cycle arrest at the G2 phase for doxorubicin in GBM43 cell lines. However, the result can vary depending on cell lines, and further experiments using other cell lines can provide better insight on the effect of the combination treatment on cell cycle.

In the apoptosis experiment, the apoptosis of cells treated with temozolomide only was similar to the control. The GBM43 cells are temozolomide-resistant cells, hence they can evade apoptosis induced by temozolomide [29]. A similar reduction in apoptosis upon treatment with temozolomide was observed for temozolomide-resistant cells by Zhu et al. (2022) [29]. However, the cells treated with the combination treatment of temozolomide and doxorubicin showed consistently higher apoptosis compared to the single treatments with temozolomide or doxorubicin. The total apoptosis for cells treated with the combination treatment was 4-times and 1.5-times higher compared to cells treated with a single treatment of temozolomide or doxorubicin, respectively. Ros et al. (2018) have observed a similar higher pro-apoptotic effect using a combination treatment with aldoxorubicin (prodrug of doxorubicin) and temozolomide compared to a single treatment with temozolomide on both temozolomide-sensitive (U87 and A172) and -resistant (T98G) cells. These results suggest that despite temozolomide not inducing apoptosis itself in temozolomide-resistant cells, combining it with doxorubicin can enhance the apoptotic effect more than the result obtained by a single treatment with doxorubicin only. One of the possible mechanisms for the higher apoptosis in combination treatment could be due to higher doxorubicin uptake by cells when combined with temozolomide. A study by Mohammad et al. (2015) has also showed higher doxorubicin uptake and higher apoptosis upon combination treatment of doxorubicin with methyl-β-cyclodextrin (MCD) [31]. In their study, the higher apoptosis for the combination treatment was due to the upregulation of proteins such as Bax and caspases 7 and 8, that enhance apoptosis, and downregulation of anti-apoptotic proteins such as Bcl-2 [31]. Further molecular study is needed to elucidate the mechanisms of higher apoptosis for the combination treatment with temozolomide and doxorubicin.

Glioblastoma is one of the most difficult-to-treat cancers due to several challenges regarding its treatment, such as disease location site, blood–brain barrier, drug resistance, etc. [2]. Surgery followed by radiation and chemotherapy with temozolomide remains the current standard of treatment; however, patient survival remains very low despite this treatment [5]. There is a pressing need to find a treatment strategy that has higher efficacy in tumor suppression and can enhance patients’ survival. In this study, we tested the effectiveness of a combination treatment of temozolomide and doxorubicin against various GBM cell lines. The findings of this study are significant as they show synergistic interaction between temozolomide and doxorubicin against both temozolomide-sensitive and -resistant cell lines. The GBM43 cells used for our study show higher expressions of O6-methylguanine-DNA methyltransferase (MGMT), a mechanism responsible for temozolomide resistance [32]. However, combination treatment of these cells with temozolomide and doxorubicin allowed for higher doxorubicin uptake and enhanced apoptosis, suggesting application of this this combination strategy to temozolomide-resistant cells as well. Doxorubicin use has its own drawbacks, such as toxicities to various organs, an inability to cross the blood–brain barrier, and drug-resistance issues. To address the blood–brain barrier issue, doxorubicin can be administered locally or in conjugation with other carriers, such as albumin, for effective delivery to the glioblastoma site [24]. Use of albumin for the effective delivery of doxorubicin was utilized by Ros et al. (2018) to deliver aldoxorubicin (albumin-binding doxorubicin) in a U87-xenograft model [24]. Further, doxorubicin can be administered locally at the surgical site of a tumor by utilizing other drug delivery systems, such as hydrogel application [21]. In our previous study, we have shown that an elastin-like polypeptide (ELP)-based hydrogel system built by combining doxorubicin-conjugated ELP and collagen allows for the sustained and tunable release of doxorubicin [21]. Hence, doxorubicin can be delivered via other delivery systems, and temozolomide can be administered orally to form a combination treatment strategy using these two drugs. Thus, our data on the synergistic interaction between these two drugs provide higher confidence in utilizing this strategy for the treatment of patients with glioblastoma. While we did not conduct specific experiments focusing on the mechanism by which dox and TMZ overcome drug resistance, we want to highlight that we observed improved outcomes when applying dox treatment to TMZ-resistant cells as part of a combination therapy. Therefore, our findings suggest that the synergy between dox and TMZ contributes to enhanced efficacy, particularly in the context of TMZ-resistant cells.

This type of combination strategy will provide advantages such as low-dose usage, multiple targets, and can address resistance issues related to each drug.

## 4. Methods

### 4.1. Effect of Combination of Temozolomide and Doxorubicin on Cell Viability

We tested the effect of the combination of temozolomide and doxorubicin on the cell viability of three different cells lines (U87, GBM43, and GBM6). The initial numbers of cells plated were 500, 1500, and 2500 for U87, GBM43, and GBM6, respectively. After 24 h of plating, the cells were treated with different concentrations of temozolomide (0, 40, 80, 160, and 320 uM), doxorubicin (0, 5, 10, 20, 40, and 80 nM), and combination of these concentrations. After 5 days of treatment, the cell viability for each treatment was measured using an MTT (3-(4,5-dimethylthiazol-2-yl)-2,5-diphenyltetrazolium bromide) assay. Briefly, MTT reagent (10 uL) was added into each well and incubated for 4 h in the dark. After 4 h, the MTT-containing media was removed and DMSO was added. The absorbance (570 nm) was measured and the percentage survivals for each treatment were determined compared to the control.

Percentage survival (treatment) = (Absorbance of Treatment (570 nM)/Absorbance for control (DMSO treated)) × 100

### 4.2. Determination of Synergy between Temozolomide and Doxorubicin

The synergy between temozolomide and doxorubicin was determined using SynergyFinder (https://synergyfinder.fimm.fi/), accessed on 12 December 2023 [22]. SynergyFinder is an online-based tool that determines synergy between a combination of drugs using various reference models, such as the highest single agent (HSA), Bliss, Loewe, and zero interaction potency (ZIP) models. The synergy scores obtained based on these models were classified as synergistic or antagonistic (synergy score < 10—antagonistic, synergy score > 10—synergistic).

### 4.3. Apoptosis

The effect of the combination treatment of temozolomide and doxorubicin on apoptosis was tested using an AlexaFluor 488 annexin V/Dead cell apoptosis kit. The GBM43 (300,000) cells were plated on a 6-well plate. After 24 h of plating, the cells were treated with temozolomide (160 uM), doxorubicin (10 nM), and the combination of temozolomide (160 uM) and doxorubicin (10 nM). The DMSO-treated and untreated cells were used as negative controls. The cells treated with etoposide (500 uM) were considered as the positive control. After 24 h of treatment, the cells were trypsinized and collected. The cells were then stained with annexin V and PI to determine the apoptotic and dead cells. Annexin V binds to the phospholipids exposed outside that are present in apoptotic cells. The PI can enter through the plasma membrane of the dead cells and stain the nucleic acid, thereby representing dead cells. The cells stained by annexin V and PI were analyzed by using flow cytometry (Novocyte by ACEA Biosciences, Inc., San Diego, CA, USA) to determine the percentages of early and late apoptotic cells.

### 4.4. Doxorubicin Uptake

The cells (100,000–200,000) were plated on a 6-well plate. After 24 h of plating, the cells were treated with different treatments (doxorubicin (10 nM), temozolomide (160 uM) + doxorubicin (10 nM), and DMSO-control + doxorubicin (10 nM)). Some wells included untreated cells that were used to subtract autofluorescence during the analysis. After 24 h of treatment, the cells were trypsinized and washed with PBS. The cells were then collected and then stained with a live/dead fixable Far Red Dead Cell Stain Kit (Invitrogen, Lane County, OR, USA). The cells were then analyzed by using flow cytometry. The live/dead stain kit stains dead cells and is detected on the APC channel. The doxorubicin has its own intrinsic fluorescence that can be detected in the PE Texas Red channel. The percentage of cells in the PE Texas Red channel but not in the APC channel was used to determine the doxorubicin uptake percentage.

### 4.5. Cell Cycle

For the cell cycle analysis, the GBM43 cells were plated on a 6-well plate, and then after 24 h of plating they were treated with either temozolomide (160 uM), doxorubicin (10 nM), or temozolomide (160 uM) + doxorubicin (10 nM). The untreated cells and DMSO treated cells were used as controls. After 48 h of treatment, cells were trypsinized and collected in flow tubes. The cells were then centrifuged at 3000 RPM for 3 min. The supernatant was removed and 200 uL PBS was added to the cell pellet. The cells were then fixed using 70% EtOH (placed on ice/30 min) and then centrifuged (3000 RPM/3 min). After removing the supernatant, the cell pellets were washed with PBS and then resuspended in 300 uL of PBS. The RNase (300 uL of 10 ng/mL) was added to the cells and incubated at RT for 5 min. After incubation, 150 uL of PI (200 ug/mL) was added and then again incubated at RT for 30 min. The cells stained with PI were then analyzed on a flow cytometer to determine the percentage of cells in each cell cycle phase (G1, S, G2).

### 4.6. Statistical Analysis

The data obtained for proliferation, doxorubicin uptake, cell cycle analysis, and apoptosis experiments were analyzed on graph prism. Two-way ANOVA was used to determine the significant differences in cell viability for cells treated with a single or combination treatment of temozolomide and doxorubicin. The doxorubicin uptake percentage for each treatment group was determined, and the autofluorescence using untreated cells was subtracted from these treatment groups (doxorubicin uptake percentage (final_treatment) = doxorubicin uptake percentage (treatment)/doxorubicin uptake (untreated)). The data obtained were then analyzed in graph prism. One-way ANOVA was used to determine the significant difference in doxorubicin uptake for different treatment groups. For apoptosis, one-way ANOVA was used to determine the significant differences in early, late, and total apoptosis between treatment groups. Further, an unpaired student t-test was used to compare the apoptosis in the cells treated with a combination of temozolomide and doxorubicin and the cells treated with its single components. One-way ANOVA was used to determine significant differences in the percentages of cells in different phases (G1, S, and G2) between the treatment groups in the cell cycle analysis.

## Data Availability

The data presented in this study are available in the article and on request from the corresponding author.
